# Antiallodynic Effects of Cannabinoid Receptor 2 (CB_2_R) Agonists on Retrovirus Infection-Induced Neuropathic Pain

**DOI:** 10.1155/2019/1260353

**Published:** 2019-07-04

**Authors:** Wen S. Sheng, Priyanka Chauhan, Shuxian Hu, Sujata Prasad, James R. Lokensgard

**Affiliations:** Neurovirology Laboratory, Department of Medicine, University of Minnesota, Minneapolis, MN 55455, USA

## Abstract

The most common neurological complication in patients receiving successful combination antiretroviral therapy (cART) is peripheral neuropathic pain. Data show that distal symmetric polyneuropathy (DSP) also develops along with murine acquired immunodeficiency syndrome (MAIDS) after infection with the LP-BM5 murine retrovirus mixture. Links between cannabinoid receptor 2 (CB_2_R) and peripheral neuropathy have been established in animal models using nerve transection, chemotherapy-induced pain, and various other stimuli. Diverse types of neuropathic pain respond differently to standard drug intervention, and little is currently known regarding the effects of modulation through CB_2_Rs. In this study, we evaluated whether treatment with the exogenous synthetic CB_2_R agonists JWH015, JWH133, Gp1a, and HU308 controls neuropathic pain and neuroinflammation in animals with chronic retroviral infection. Hind-paw mechanical hypersensitivity in CB_2_R agonist-treated versus untreated animals was assessed using the MouseMet electronic von Frey system. Multicolor flow cytometry was used to determine the effects of CB_2_R agonists on macrophage activation and T-lymphocyte infiltration into dorsal root ganglia (DRG) and lumbar spinal cord (LSC). Results demonstrated that, following weekly intraperitoneal injections starting at 5 wk p.i., JWH015, JWH133, and Gp1a, but not HU308 (5 mg/kg), significantly ameliorated allodynia when assessed 2 h after ligand injection. However, these same agonists (2x/wk) did not display antiallodynic effects when mechanical sensitivity was assessed 24 h after ligand injection. Infection-induced macrophage activation and T-cell infiltration into the DRG and LSC were observed at 12 wk p.i., but this neuroinflammation was not affected by treatment with any CB_2_R agonist. Activation of JAK/STAT3 has been shown to contribute to development of neuropathic pain in the LSC and pretreatment of primary murine microglia (2 h) with JWH015-, JWH133-, or Gp1a-blocked IFN-gamma-induced phosphorylation of STAT1 and STAT3. Taken together, these data show that CB_2_R agonists demonstrate acute, but not long-term, antiallodynic effects on retrovirus infection-induced neuropathic pain.

## 1. Introduction

Approximately 35–50% of HIV-1 patients undergoing successful combination antiretroviral therapy (cART) experience peripheral neuropathic pain, which currently makes it the most common neurological complication of infection [1–4]. Our laboratory has been investigating the role of activated, brain-infiltrating peripheral immune cells in driving chronic activation of brain-resident microglia following viral infection, particularly through production of IFN-*γ* [5, 6]. Studies demonstrate that this type of immune-induced activation of resident glial cells is also emerging as a common mechanism underlying various types of chronic pain, reviewed in [7]. Despite its clinical significance, neuropathic pain in the context of well-controlled HIV infection is still an understudied area, in part because of the lack of convenient animal models. Correspondingly, data from us and others show that distal symmetric polyneuropathy (DSP) develops in animals with murine acquired immunodeficiency syndrome (MAIDS) by 6–8 wk following infection with the LP-BM5 retrovirus mixture [8, 9].

Chronic viral infection and infection-induced inflammation are known to induce production of neurotoxic mediators which have been associated with DSP, reviewed in [10]. While HIV itself does not replicate in neurons, neuropathological studies have demonstrated the presence of proviral DNA, mRNA, and p24 antigen within macrophages in dorsal root ganglia (DRG) of HIV-infected patients [11]. A consistent neuropathological abnormality in DSP patients is the presence of activated macrophages in the DRG, which express MHCII antigens and proinflammatory cytokines [12]. In many forms of DSP, the disease correlates well with the degree of macrophage infiltration and microglial cell activation [13, 14]. Numerous studies have demonstrated a critical role of activated microglia [15–18], as well as astrocytes [19–21] and satellite glial cells in the DRG [22–25], in the development of neuropathic pain.

At the present time, there are no FDA-approved pharmacologic agents available which are specifically designed for treatment of chronic HIV-associated neuropathy. The analgesics currently used (i.e., opioids) are only modestly effective and possess significant CNS side effects [26, 27]. Derivatives of *Cannabis sativa* have frequently been used as analgesics, and links between cannabinoids and peripheral neuropathy have been established in animal models using nerve transection, chemical-induced pain, and various other stimuli [28–32]. It is well known that diverse types of peripheral neuropathic pain respond differently to standard drug intervention [33, 34], but little is currently known regarding the effects of inflammatory modulation through cannabinoid receptor 2 in the context of peripheral neuropathy due to chronic retroviral infection.

There are two distinct types of cannabinoid receptors: CB_1_Rs which are expressed primarily in neurons and CB_2_Rs which are found mainly in cells of the immune system and glia [35, 36]. So, exploring potential therapies centered on CB_2_R-specific agonists has the potential benefit of more specifically targeting immune cell activation while simultaneously avoiding unwanted, psychoactive side effects associated with activating CB_1_Rs. CB_2_R activation clearly results in suppressive effects such as reduced cytokine release and inhibition of immune cell chemotaxis [37]. So, CB_2_Rs have enormous potential for pharmacological manipulation, but because of the sheer diversity of their physiological influences, huge challenges also exist. Although an abundance of experimental data demonstrate the effectiveness of cannabinoids as potentially effective analgesics, it has been difficult to translate the use of cannabis extracts into clinically effective therapies. Overall, to date, results from reported clinical trials do not make a compelling argument for their use in various types of neuropathic pain, recently reviewed in [38]. Nevertheless, new approaches which lead to development of more specific and effective analgesics for neuropathic pain are desperately needed, and immunomodulation through the CB_2_R system is clearly a promising strategy. The application of more specific, synthetic CB_2_R agonists may be one approach to increase the clinical efficacy of cannabinoid therapy.

Although not a perfect representation of human AIDS, the MAIDS (i.e., LP-BM5 infection) model in mice does allow us to investigate mechanisms that drive neuropathic pain during chronic retroviral infection. In a previous study, we have reported neuroinflammation and nitrosative damage associated with peripheral neuropathy in this murine model, but no drugs or therapeutic treatment interventions were used [9]. In experiments presented here, we follow up to our previous study by testing the hypothesis that treatment using synthetic CB_2_R agonists can control the neuropathic pain and limit the neuroinflammation seen during chronic retroviral infection.

## 2. Materials and Methods

### 2.1. Ethical Approval

The animal care protocol employed was approved by our Institutional Animal Care and Use Committee (Protocol Number 1709-35110A). Animal well-being was monitored according to Research Animal Resources (RAR) procedures. All efforts were made to reduce animal suffering, and when required, mice were euthanized using isoflurane inhalation.

### 2.2. Reagents

Chemicals were from Sigma-Aldrich (St. Louis, MO); fetal bovine serum was from Peak Serum (Wellington, CO); DNase was from Life Technologies (Carlsbad, CA); CB_2_R agonists: JWH015, JWH133, Gp1a, and HU308, were from Tocris Bioscience (Minneapolis, MN).

### 2.3. Virus and Animals

LP-BM5 was acquired from the NIH AIDS Reagent Program (Germantown, MD, USA). Stocks of virus were prepared as cell-free supernatants of SC-1 cells, as described previously [6]. The XC plaque assay for BM5_eco_ was used to determine viral titers. Female C57BL/6 mice were obtained from The Jackson Laboratory, Bar Harbor, ME, USA, and were housed at the RAR facility. Mice were infected via intraperitoneal (i.p.) injection (2 × 10^4^/PFU dose/2 doses with 3 d between doses) of the LP-BM5 retrovirus mixture [6].

### 2.4. Assessment of Mechanical Allodynia

The MouseMet electronic von Frey system (Topcat Metrology, Cambridge, UK) was used to assess both left and right hind-paw mechanical hypersensitivity weekly between 5 and 12 wk following LP-BM5 infection. Mice were acclimated to the testing chambers for 20 min before testing. Bilateral measurements of six sets were performed for each mouse, and data were calculated as the paw withdrawal threshold in grams of force.

### 2.5. Isolation of Leukocytes from Lumbar Spinal Cord (L3–L5) and Dorsal Root Ganglia for Flow Cytometry

Tissue mononuclear cells were isolated from the lumbar spinal cord (LSC) of uninfected, infected, and infected, CB_2_R agonist-treated animals using procedures described previously [39, 40]. Mice were anesthetized with ketamine/xylazine and transcardial perfused with saline before tissue collection. The LSCs were dissected (*n*=4–6) and finely minced in RPMI 1640 at RT. Preparations of single cells were suspended in 30% Percoll and banded on a 70% Percoll cushion. Leukocytes obtained at the 30–70% Percoll interface were harvested and counted. A nonenzymatic dissociation method was employed to isolate mononuclear cells from DRG, as described previously [41]. Six ganglia (L3–L5) per mouse were collected (*n*=4–6 animals/group/experiment). DRGs were homogenized using a 1 ml syringe attached with a 21 G needle and then a 23 G needle, filtered through a cell strainer, and counted as described previously.

Isolated cells were treated with Fc Block (i.e., anti-CD32/CD16) to inhibit nonspecific Ab binding. Cells were stained with anti-immune surface markers (i.e., anti-MHC-II-PE (clone M5/114.15.2; cat# 12-5321-82; eBioscience, San Diego, CA, USA), anti-CD11b-AF700 (clone M1/70; cat# 56-0112-82; eBioscience), anti-CD45-PE-Cy5 (clone 30-F11; cat# 15-0451-81; eBioscience), anti-CD4-BV510 (clone GK1.5; cat# 100449; BioLegend, San Diego, CA, USA), and anti-CD8-PE-Cy7 (clone 53-6.7; cat# 25-0081-82; eBioscience)). Counts were acquired (10^5^/sample) using an LSR flow cytometer (BD Biosciences, San Jose, CA), and the FACSDIVA software was used to calculate the absolute cell number. The FlowJo software (Tree Star, Ashland, OR, USA) was used to analyze the data.

### 2.6. Microglial Cell Cultures

Cerebral cortical cells were collected from one-day-old C57BL/6 pups followed by cell dissociation using trypsin (0.25% in HBSS) for 30 min. Cells were centrifuged and washed 3 times in HBSS, suspended in DMEM with FBS (10%), Fungizone® (250 pg/mL), gentamicin (50 *μ*g/mL), penicillin (100 U/mL), and streptomycin (100 *μ*g/mL), and seeded onto Falcon tissue culture flasks (75 cm^2^). Culture media were changed at d 1 and d 4. Microglial cells were harvested at d 12 and plated onto 12-well Falcon cell culture plates in DMEM with FBS (6%) for 60 min at 37°C. Cell culture media were replaced with DMEM without FBS overnight, followed by pretreatment with CB_2_R agonists for 2 h prior to IFN-*γ* stimulation for 20 min.

### 2.7. Western Blotting

Following treatment, microglial culture media were removed and cells were lysed with the 2x Laemmli sample buffer containing 5% 2-mercaptoethanol. The samples were then heated at 100°C for 5 min and vortexed before being loaded onto 4–15% precast acrylamide gels for electrophoresis. Gels were transblotted onto nitrocellulose membranes (0.45 *µ*m), rinsed in TTBS (Tris-HCl with NaCl and Tween 20), and blocked with 5% Blotto (Santa Cruz, Dallas, TX) for 1 h at RT. Membranes were then probed with rabbit anti-*β*-actin (#4970) and rabbit anti-phospho-Stat3_(Tyr705)_ (#9131) or -Stat1_(Tyr701)_ (#9171) Abs (Cell Signaling Technology, Danvers, MA) overnight at 4°C. After washing 3x with TTBS, alkaline phosphatase (AP) conjugated secondary antibody (1 : 5,000 in 1% Blotto, Promega, Madison, WI) was added to the membranes at RT for 1 h. Following 3x washing with TTBS, membranes were rinsed in the assay buffer (1x) twice (2 min each) and then incubated in the substrate solution (CDP-Star, Applied Biosystems, Foster City, CA) for 10 min followed by imaging using an Odyssey Imaging System (LI-COR Biosciences, Lincoln, NE).

### 2.8. Statistical Analysis

One-way ANOVA with Fisher's LSD post hoc test was used for the analysis of behavioral testing. ANOVA with Tukey's test was employed for graphical analysis. Significant differences were obtained when *p* < 0.05.

## 3. Results

### 3.1. Acute Antiallodynic Effects of CB_2_R Agonists on Retrovirus-Infected Animals

**Figure 1:**
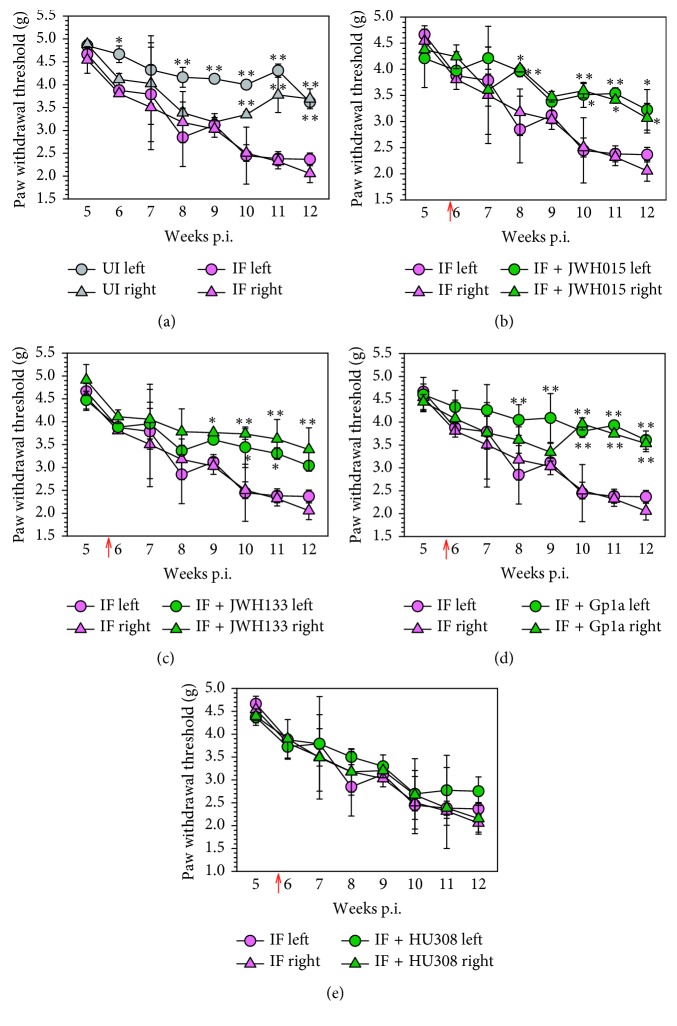
Acute antiallodynic effects of CB_2_R agonists on retrovirus-infected animals. (a) C57BL/6 mice were randomly assigned into uninfected (UI) or LP-BM5-infected (IF) groups (*n*=8/group), and hind-paw mechanical hypersensitivity was assessed weekly via the MouseMet electronic von Frey system between 5 and 12 wk p.i. Following LP-BM5 infection, synthetic CB_2_R ligands were injected (5 mg/kg, i.p.) once/wk starting at 5 wk·p.i. (red arrow). Assessment of allodynia was performed weekly 2 h after injection of the indicated ligand: (b) JWH015; (c) JWH133; (d) Gp1a; (e) HU308. Data are presented as the mean ± SE paw withdrawal threshold with *n*=8/group. ^*∗*^*p* < 0.05 and ^*∗∗*^*p* < 0.01 vs. corresponding paw/wk p.i. (ANOVA with Fisher's LSD post hoc analysis).

C57BL/6 mice infected with the LP-BM5 retrovirus mixture have previously been reported to show symptoms of DSP by 6 wk p.i. [8, 9]. Here, we evaluated whether treatment with the exogenous synthetic CB_2_R agonists: JWH015, JWH133, Gp1a, and HU308, could control neuropathic pain and neuroinflammation in animals with this chronic retroviral infection. Using the MouseMet electronic von Frey system, we assessed hind-paw mechanical hypersensitivity in CB_2_R agonist-treated versus untreated animals. We were first able to once again repeat findings that mice infected with LP-BM5 exhibited mechanical hind-paw hypersensitivity by 6 wk p.i., with no difference between left and right paws (Figure 1(a)). Additional results showed that weekly intraperitoneal injections (5 mg/kg) starting at 5 wk p.i. of JWH015 (Figure 1(b)), JWH133 (Figure 1(c)), and Gp1a (Figure 1(d)), but not HU308 (Figure 1(e)), significantly ameliorated allodynia when assessed 2 h after injection of the CB_2_R agonist. Although viral loads were found to persist within DRG and LSC tissues, no differences in either BM5_def_ or BM5_eco_ were detected between CB_2_R agonist-treated and untreated groups when assessed using real-time PCR at 12 wk p.i. (data not shown), as described in [9].

### 3.2. Lack of Long-Term Antiallodynic Effects of CB_2_R Agonists on Hind-Paw Mechanical Hypersensitivity

**Figure 2:**
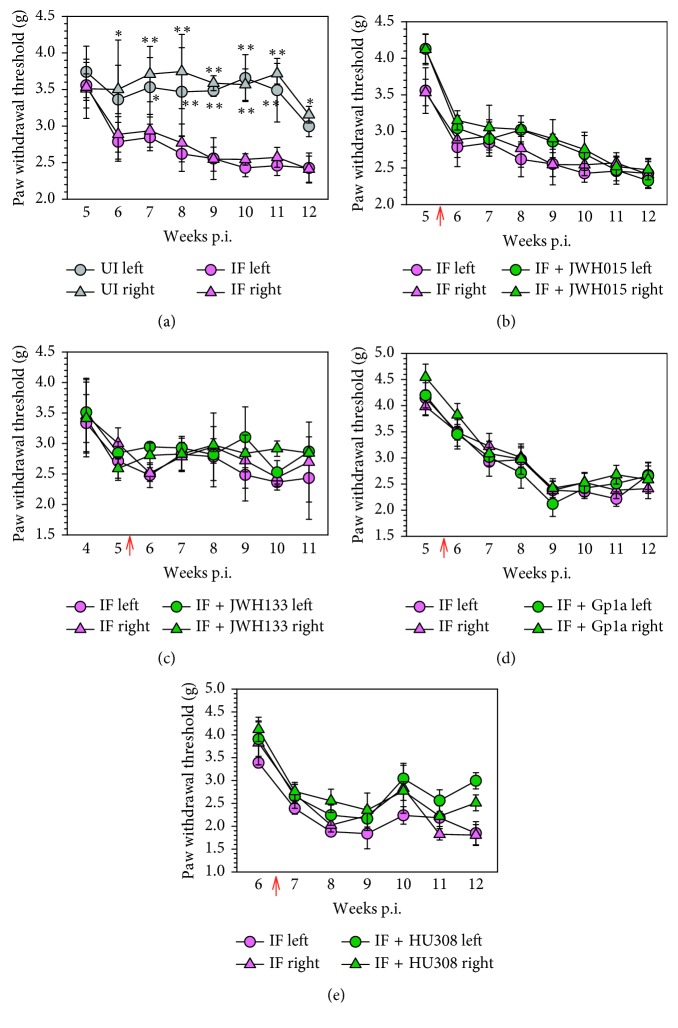
Lack of long-term antiallodynic effects of CB_2_R agonists on hind-paw mechanical hypersensitivity. (a) LP-BM5-infected mice were randomly assigned into uninfected (UI) or infected (IF) groups (*n*=8/group), and hind-paw mechanical hypersensitivity was assessed weekly via the MouseMet electronic von Frey system between 5 and 12 wk p.i. After LP-BM5 infection, synthetic CB_2_R ligands were injected (5 mg/kg, i.p.) twice/wk starting at 5 or 6 wk (HU308) p.i. (red arrow). Assessment of hind-paw mechanical hypersensitivity was performed weekly 24 h after first injection of the indicated CB_2_R ligand: (b) JWH015; (c) JWH133; (d) Gp1a; (e) HU308. Data are presented as the mean ± SE paw withdrawal threshold with *n*=8/group. ^*∗*^*p* < 0.05 and ^*∗∗*^*p* < 0.01 vs. corresponding paw/wk p.i. (ANOVA with Fisher's LSD post hoc analysis).

Our next experiments examined the duration of the antiallodynic effect following CB_2_R agonist treatment. In a parallel experiment, we again established that mice infected with LP-BM5 exhibited mechanical hind-paw hypersensitivity (Figure 2(a)). Interestingly, when the same CB_2_R agonists were injected twice per week starting at 5 wk p.i. (6 wk for HU308) and mechanical sensitivity was assessed 24 h after ligand injection, treatment with JWH015 (Figure 2(b)), JWH133 (Figure 2(c)), Gp1a (Figure 2(d)), or HU308 (Figure 2(e)) was not found to display significant antiallodynic effects. Likewise, animals treated with vehicle alone (i.e., 10% DMSO/10% TWEEN 80 in saline) did not display analgesic effects.

### 3.3. Effects of CB_2_R Agonist Treatment on Macrophage and T-Cell Infiltration into the DRG of LP-BM5-Infected Animals

**Figure 3:**
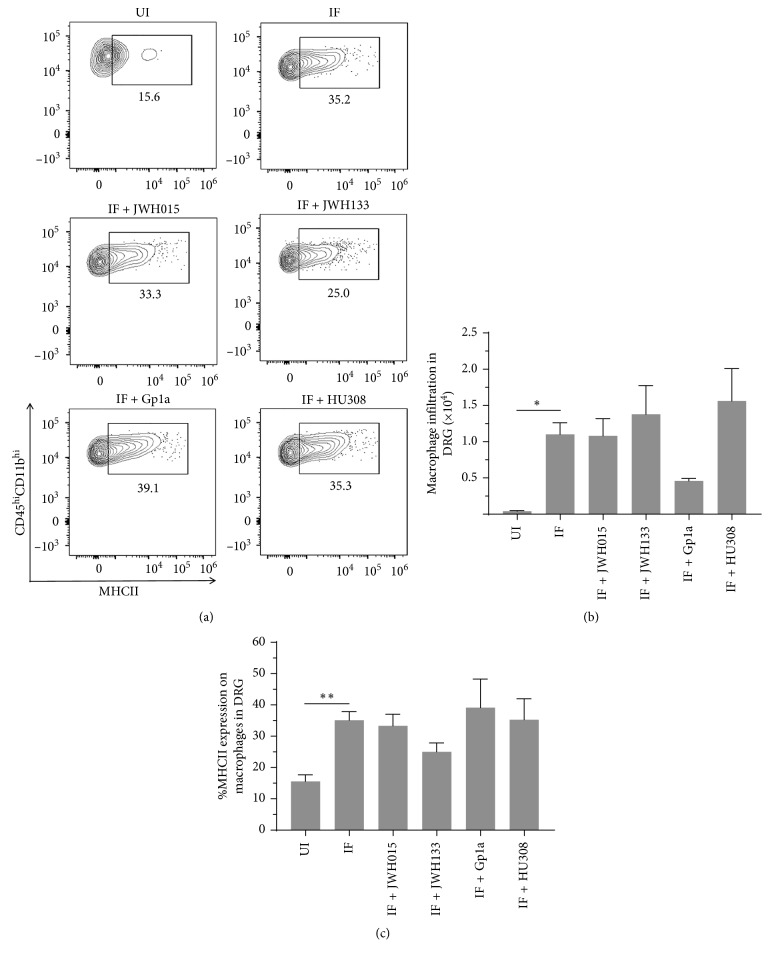
Effects of CB_2_R agonist treatment on macrophage infiltration into the DRG of LP-BM5-infected animals. After LP-BM5 infection, synthetic CB_2_R ligands were injected (5 mg/kg, i.p.) twice/wk starting at 5 or 6 wk (HU308) p.i. At 12 wk p.i., DRG were dissected and mononuclear cells from six ganglia (L3–L5) per mouse were isolated using nonenzymatic dissociation. Isolated cells were then stained using Abs specific for CD45, CD11b, and MHCII for analysis by flow cytometry. (a) Representative contour plots show percentages of macrophages (i.e., CD45^hi^CD11b^hi^) expressing MHCII within uninfected (UI), infected (IF), and infected (IF + indicated CB_2_R ligand), agonist-treated DRG. (b) Numbers of macrophages infiltrating DRG of animals belonging to the indicated treatment groups. (c) Frequency of macrophages expressing MHCII within DRG of animals belonging to the indicated groups. Pooled data present absolute numbers (mean ± SE) from two independent experiments using 4–6 animals/group. ^*∗*^*p* < 0.05; ^*∗∗*^*p* < 0.01.

**Figure 4:**
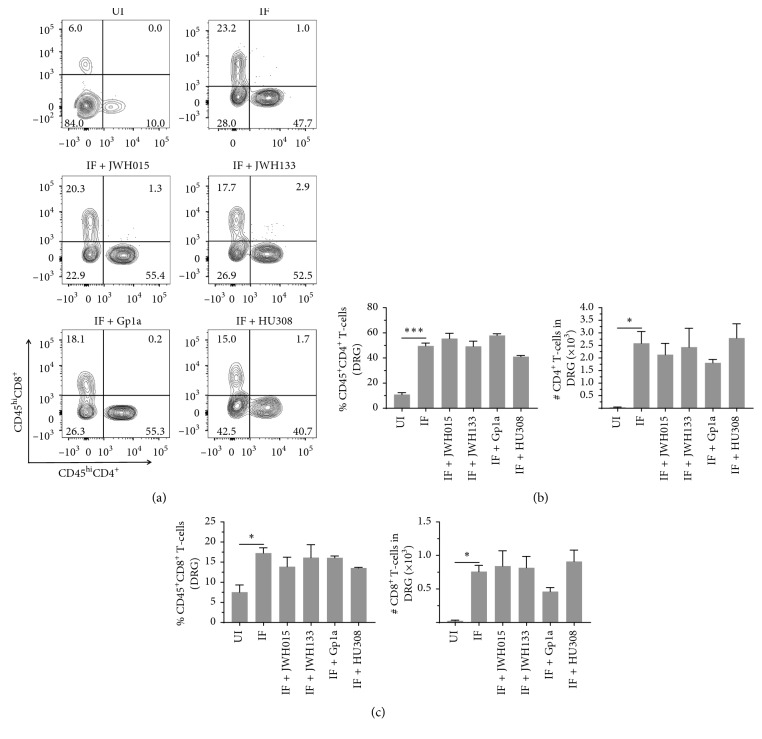
Effects of CB_2_R agonists on lymphocyte infiltration into the DRG. After LP-BM5 infection, synthetic CB_2_R ligands were injected (5 mg/kg, i.p.) twice/wk starting at 5 or 6 wk (HU308) p.i. At 12 wk p.i., DRG were dissected and mononuclear cells from six ganglia (L3–L5) per mouse were isolated using nonenzymatic dissociation. Mononuclear cells were then labeled with Abs specific for CD45, CD4, and CD8 for analysis by flow cytometry. (a) Representative contour plots show percentages of CD4^+^ and CD8^+^ T-lymphocytes within uninfected (UI), infected (IF), and infected (IF + indicated CB_2_R ligand), agonist-treated DRG. (b) Frequency and number of CD4^+^ T-cells infiltrating into DRG of animals belonging to the indicated treatment groups. (c) Frequency and number of CD8^+^ T-cells infiltrating into DRG of animals belonging to the indicated treatment groups. Pooled data present absolute numbers (mean ± SE) of CD4^+^ and CD8^+^ T-cells from two independent experiments using 4–6 animals per group. ^*∗*^*p* < 0.05; ^*∗∗∗*^*p* < 0.001.

We have recently reported inflammation of the DRG in LP-BM5-infected mice, as assessed by increased infiltration of CD45^hi^CD11b^+^ macrophages, as well as CD4^+^ and CD8^+^ T-cells, concomitant with development of DSP [9]. Here, we went on to use multicolor flow cytometry to determine the effects of CB_2_R agonist treatment on macrophage infiltration into DRG and cellular activation, as assessed by upregulation of MHCII (Figure 3(a)). We observed significant differences in macrophage numbers (Figure 3(b)) and MHCII expression (Figure 3(c)) between uninfected (UI) and infected (IF) animals when assessed at 12 wk p.i. However, this macrophage-induced neuroinflammation was not found to be affected by treatment with any CB_2_R agonist (Figure 3). An apparent decrease in macrophage infiltration was observed following treatment with Gp1a, but this difference was not statistically significant. Likewise, significant CD4^+^ and CD8^+^ T-lymphocyte infiltration into the DRG was observed in infected mice at 12 wk p.i., when compared to uninfected animals (Figure 4(a)). Similar to the situation with macrophages, this T-cell neuroinflammation was not affected by treatment with any CB_2_R agonist. Neither the percentage nor the number of CD4^+^ (Figure 4(b)) or CD8^+^ (Figure 4(c)) T-cells within the DRG-infiltrating CD45^hi^ leukocyte population was affected by treatment with CB_2_R agonists.

### 3.4. Effects of CB_2_R Agonist Treatment on Macrophage and T-Cell Infiltration into the LSC of LP-BM5-Infected Animals

**Figure 5:**
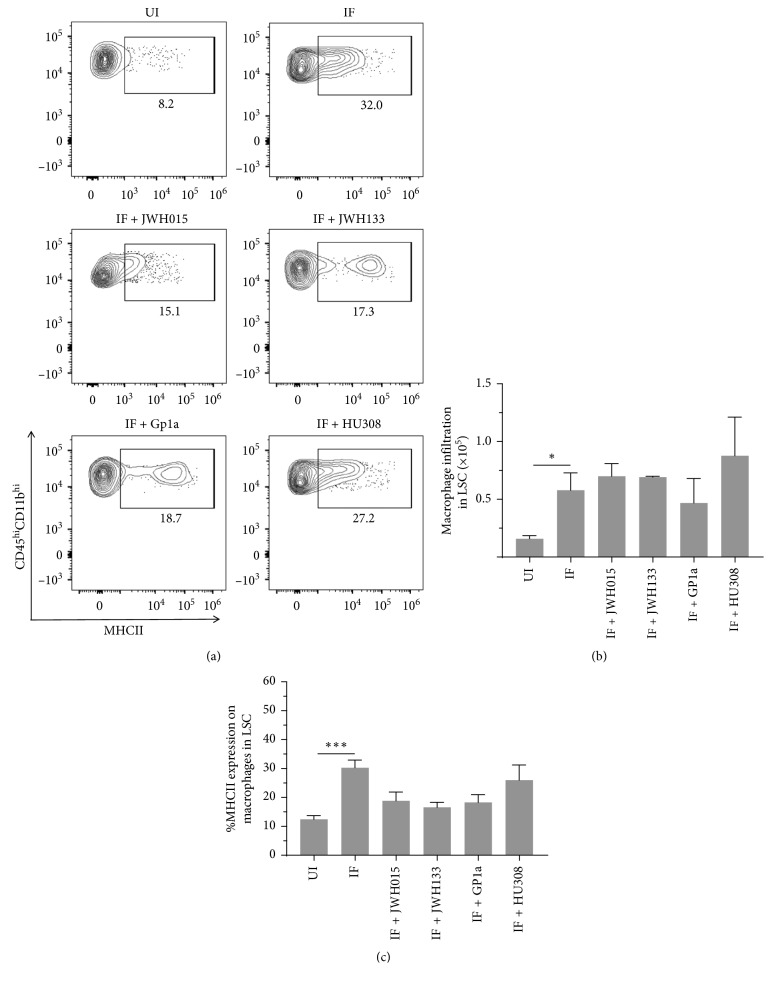
Effects of CB_2_R agonist treatment on macrophage infiltration into the LSC of LP-BM5-infected animals. After LP-BM5 infection, synthetic CB_2_R ligands were injected (5 mg/kg, i.p.) twice/wk starting at 5 or 6 wk (HU308) p.i. At 12 wk p.i., LSCs were dissected and infiltrating leukocytes were banded on a 30–70% Percoll cushion, collected, and labeled with Abs specific for CD45, CD11b, and MHCII for analysis by flow cytometry. (a) Representative contour plots show the percentages of macrophages (i.e., CD45^hi^CD11b^hi^) expressing MHCII within uninfected (UI), infected (IF), and infected (IF + indicated CB_2_R ligand), agonist-treated LSC. (b) Numbers of macrophages infiltrating into the LSC of animals belonging to the indicated treatment groups. (c) Frequency of macrophages expressing MHCII within the LSC of animals belonging to the indicated groups. Pooled data present absolute numbers (mean ± SE) from two independent experiments using 4–6 animals/group. ^*∗*^*p* < 0.05; ^*∗∗∗*^*p* < 0.001.

**Figure 6:**
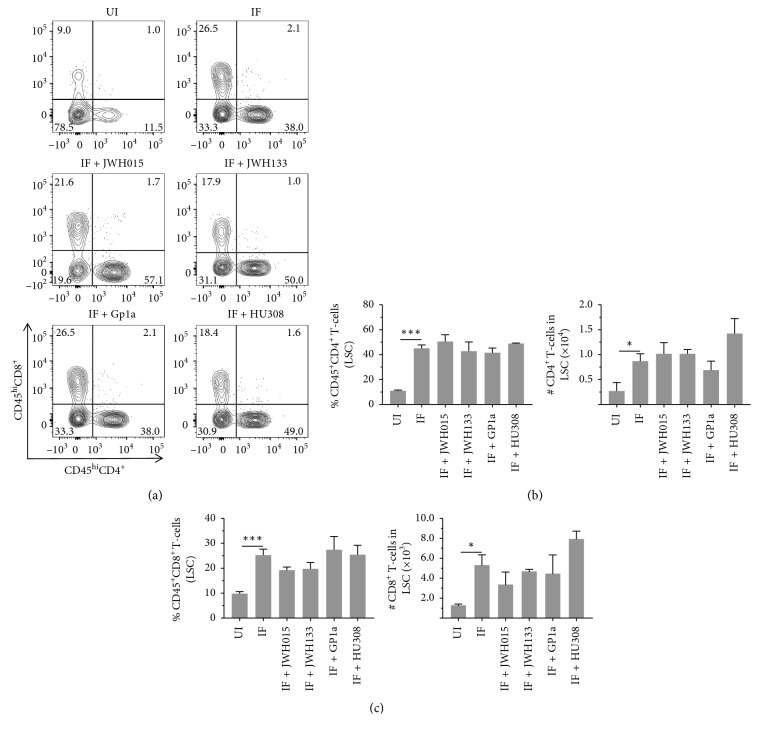
Effects of CB_2_R agonist treatment on T-lymphocyte infiltration into the LSC of LP-BM5-infected animals. After LP-BM5 infection, synthetic CB_2_R ligands were injected (5 mg/kg, i.p.) twice/wk starting at 5 or 6 wk (HU308) p.i. At 12 wk p.i., LSCs were dissected and infiltrating leukocytes were banded on a 30–70% Percoll cushion, collected, and labeled with Abs specific for CD45, CD4, and CD8 for analysis by flow cytometry. (a) Representative contour plots show percentages of CD4^+^ and CD8^+^ T-lymphocytes within uninfected (UI), infected (IF), and infected (IF + indicated CB_2_R ligand), agonist-treated LSC. (b) Frequency and number of CD4^+^ T-cells infiltrating into the LSC of animals belonging to the indicated treatment groups. (c) Frequency and number of CD8^+^ T-cells infiltrating into the LSC of animals belonging to the indicated treatment groups. Pooled data present absolute numbers (mean ± SE) of CD4^+^ and CD8^+^ T-cells from two independent experiments using 4–6 animals per group. ^*∗*^*p* < 0.05; ^*∗∗∗*^*p* < 0.001.

Multicolor flow cytometry was also used to determine the effects of CB_2_R agonists on macrophage and T-lymphocyte infiltration into the LSC. Here, infection-induced infiltration of CD45^hi^CD11b^+^ macrophages was observed at 12 wk p.i. (Figure 5(a)). Significant differences in macrophage numbers (Figure 5(b)) and MHCII expression (Figure 5(c)) were detected between uninfected (UI) and infected (IF) animals when assessed at 12 wk p.i., but again this macrophage inflammation was found not to be affected by treatment with any CB_2_R agonist (IF + indicated CB_2_R agonist). Likewise, significant CD4^+^ and CD8^+^ T-lymphocyte infiltration into the LSC was observed within infected mice at 12 wk p.i., when compared to uninfected animals (Figure 6(a)). Similar to the situation with macrophages, this T-cell neuroinflammation was not affected by treatment with any CB_2_R agonist. The percentage and number of CD4^+^ (Figure 6(b)) or CD8^+^ (Figure 6(c)) T-cells within the LSC-infiltrating CD45^hi^ leukocyte population were not found to be affected by treatment with CB_2_R agonists.

### 3.5. CB_2_R Agonists Dampen STAT3/STAT1 Activation in Microglia

**Figure 7:**
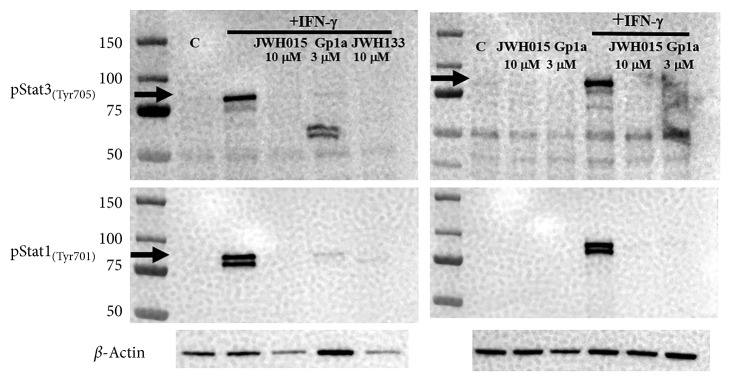
CB_2_R agonists dampen STAT3 and STAT1 activation in primary microglial cells. Primary murine microglial cultures were left untreated or treated with the indicated CB_2_R ligands: JWH015 (10 *μ*M), Gp1a (3 *µ*M), or JWH133 (10 *µ*M), for 2 h followed by IFN-*γ* stimulation for 20 min. Cell lysates were collected in the 2x sample buffer, electrophoresed, transblotted, and probed for *β*-actin and phosphorylated STAT3_(Tyr705)_ or STAT1_(Tyr701)_.

It has been reported that STAT3 contributes to chronic pain following nerve injury [42]. The activity of STAT3 is significantly enhanced following peripheral nerve damage [43], and blocking its activation ameliorates mechanical allodynia [42]. For this reason, we went on to examine the effect of pretreatment with CB_2_R agonists on IFN-*γ*-induced STAT3/STAT1 phosphorylation in primary murine microglia. We examined whether pretreatment of primary murine microglial cells (2 h) with JWH015, JWH133, or Gp1a could block IFN-*γ*-induced phosphorylation of STAT3/STAT1. In these studies, we found that treatment of murine microglia with IFN-*γ* (20 min) clearly induced STAT3/STAT1 phosphorylation, and pretreatment of the cultures with JWH015 (10 *μ*M), JWH133 (10 *µ*M), or Gp1a (3 *µ*M) for 2 h prior to IFN-*γ* stimulation dampened activation of this pathway (Figure 7).

## 4. Discussion

CB_2_Rs have been shown to be expressed at the leading edge of activated microglia [44], and their specific engagement is well known to suppress multiple cellular responses [45]. In addition to effects on macrophages and microglia, CB_2_R agonists have also been shown to inhibit T-cell function including cytokine production, cellular migration, proliferation, and, more recently, mixed lymphocyte reactions (MLRs) [46–50]. In previous studies, we have found inhibition of HIV-1 p24 expression in primary human microglia and CD4^+^ T-cells by the synthetic, nonselective CB_1_R and CB_2_R agonists WIN55,212-2 and CP55,940 was mediated primarily through CB_2_Rs [51, 52]. These same agents also suppressed production of proinflammatory mediators such as nitric oxide (NO) by human astrocytes [53]. We have also reported that WIN55,212-2 and JWH015 blunted HIV-1 gp120-induced damage to dopaminergic neurons, as measured by dopamine transporter (DAT) function (viz. ^3^H-DA uptake), apoptosis (viz. DNA fragmentation), and lipid peroxidation (viz. 8-isoprostane), and that these effects were mediated through actions by CB_2_Rs on microglial cells [54].

Previous studies demonstrate that animals begin to display hind-paw mechanical hypersensitivity approximately 5 to 6 wk following infection with the LP-BM5 retrovirus mixture [8, 9] and that viral infection is well established at this time point. For this reason, we initiated weekly i.p. injections of the selective, synthetic CB_2_R agonists: JWH015, JWH133, Gp1a, and HU308, at this time point p.i. As in a number of other preclinical studies where cannabinoids were found to be effective in models of neuropathic pain, reviewed in [55], we found that 3 of the ligands (JWH015, JWH133, and Gp1a), but not HU308, were effective in reducing mechanical allodynia induced by chronic retroviral infection when assessed 2 h following their injection (Figure 1). While many earlier cannabinoid studies implicated a CB_1_R-mediated analgesic action, the effectiveness of selective CB_2_R agonists has also been reported in neuropathic pain models [56, 57]. Specifically, CB_2_R is recognized as key for paclitaxel chemotherapy-induced neuropathic pain [58, 59]. At this time, it is unknown why HU308 displayed a different effect when compared with the other three CB_2_R agonists, but functional selectivity by which different cannabinoid agonists possess differential effects (e.g., agonist, inverse agonist, or antagonist), mode of binding (e.g., orthosteric or allosteric), and activation of signal transduction pathways (e.g., adenylate cyclase or MAP kinase) has been described for CB_2_R ligands [60]. Factors such as these may play a role in the differential effect exhibited by HU308. Finally, no direct effect on viral load was detected following treatment with any of the CB_2_R agonists, as assessed using real-time PCR at 12 wk p.i. performed as previously described [9].

Neuropathic pain is a response to damage, and we have previously detected elevated levels of 3-nitrotyrosine within the LSC and DRG of LP-BM5-infected animals, an indicator of nitric oxide- (NO-) induced protein damage [9]. 3-Nitrotyrosine was observed in both IB4^+^ small and NF200^+^ large DRG sensory neurons. Increasing attention is being focused on nonneuronal mechanisms involving immune cells that may amplify or resolve chronic pain. Because of the well-characterized anti-inflammatory and neuroprotective effects of synthetic CB_2_R agonists, in another set of experiments, we hypothesized that these agonists would control chronic, damage-induced pain. However, this hypothesis was not supported. When the same CB_2_R agonists were injected twice per week starting at 5 or 6 wk p.i. and antiallodynic responses were assessed 24 h after ligand injection, neither JWH015, JWH133, Gp1a, nor HU308 displayed significant long-term antiallodynic effects (Figure 2).

We have previously reported that LP-BM5 infection drives CD45^hi^CD11b^+^ macrophages as well as CD4^+^ and CD8^+^ T-cell neuroinflammation in the DRG of infected animals [9]. These findings were confirmed here, where significantly more leukocytes were found within ganglia of infected animals. Interestingly, treatment with any of the CB_2_R agonists did not inhibit leukocyte migration *in vivo*, when tissues were harvested at 12 wk p.i. (Figures 3 and 4). A similar situation was observed when leukocyte infiltration into the spinal cord was examined. When LSCs were examined using multicolor flow cytometry at 12 wk p.i., macrophage and T-cell infiltration into tissues of infected animals was observed, but CB_2_R agonist treatment had no inhibitory effects on chemotaxis (Figures 5 and 6). Given the well-recognized effects of CB_2_R agonists on cellular migration [48, 49, 61], our lack of chemotaxis inhibition was surprising.

Although mechanisms responsible for the acute effects of CB_2_R agonist treatment remain to be determined, because they did not produce long-lasting antiallodynic effects or affect infection-induced immune cell infiltration into the DRG or LSC, it appears unlikely that the short-term effects we observed 2 h after injection were due to modulation of immune-mediated tissue damage. More likely, the observed short-term antiallodynia resulted from CB_2_R-mediated interference with signal transduction pathways and the blockade of cytokine release associated with the response. Proinflammatory cytokines produced by CNS-infiltrating T-lymphocytes are well known to activate resident glial cells. In the feline immunodeficiency virus (FIV) model of DSP, STAT1 and iNOS have been demonstrated to be responsible for neuroinflammation-induced damage to DRG neurons [52]. Because TNF-*α*- and IL-1*β*-induced STAT3 activation in the DRG has also been reported to mediate mechanical allodynia following oxaliplatin treatment [62], we investigated the effects of the synthetic CB_2_R agonists on STAT3. These studies used cultures of primary murine microglia along with a 2 h pretreatment with JWH015, JWH133, or Gp1a (i.e., the agonists which displayed short-term antiallodynic effects). Results obtained from these studies showed that CB_2_R agonist treatment potently blocked IFN-*γ*-induced STAT1 and STAT3 phosphorylation (Figure 7).

## 5. Conclusions

Data presented here show that synthetic CB_2_R agonists demonstrate acute, but not long-term, antiallodynic effects on retrovirus infection-induced neuropathic pain.

## Data Availability

The data used to support the findings of this study are included within the article.
